# Relation between Environmental Factors and Children’s Health Behaviors Contributing to the Occurrence of Diet-Related Diseases in Central Poland

**DOI:** 10.3390/ijerph16010052

**Published:** 2018-12-26

**Authors:** Katarzyna Zadka, Ewelina Pałkowska-Goździk, Danuta Rosołowska-Huszcz

**Affiliations:** Department of Dietetics, Faculty of Human Nutrition and Consumer Sciences, Warsaw University of Life Sciences WULS-SGGW, 159c Nowoursynowska Str., 02-776 Warsaw, Poland; ewelina_palkowska_gozdzik@sggw.pl (E.P.-G.); danuta_rosolowska_huszcz@sggw.pl (D.R.-H.)

**Keywords:** childhood behaviors, diet, physical activity, chronic disease, social determinants, overweight, mother education influence, place of residence influence, income influence

## Abstract

Proper nutrition is a key element in maintaining normal body weight in children and one of the most important factors influencing their optimum development, growth, and health in the future. The aim of this cross-sectional study was to identify environmental factors which affect health behaviors of children aged 7–14, contributing to the occurrence of diet-related diseases. The study was performed with participation of 892 school children. The investigated environmental factors were as follows: the place of residence, maternal education level, and the level of family income. A questionnaire was used to collect the data from mothers. The study indicated a prevalence of overweight and obesity among children of 13.9% and 1.2%, respectively. Being underweight applied to 20.0% of examined children, more often to girls. In terms of nutritional behaviors an insufficient consumption of vegetables, fruit, whole grain products, dairy products, and fish was observed. The frequency of vegetable and fruit consumption significantly decreased with an increase in child’s body mass index (BMI) (G = −0.110, *p* < 0.05 and G = −00.114, *p* < 0.05). Overall, 29.7% of children devoted less than 30 minutes a day to physical activity, besides the physical education classes at school. Boys were more often physically active than girls (G = 0.205, *p* < 0.0005). There was a positive correlation between frequency of vegetable (G = 0.167, *p* < 0.0005) and fruit (G = 0.155, *p* < 0.005) consumption and mothers’ education level. Girls ate fruit more frequently than boys (G = 0.116, *p* < 0.05). Higher family income was associated with more frequent whole grain consumption (G = 0.095, *p* < 0.05), while living in the city was negatively correlated with activity (G = −0.121, *p* < 0.05) and dairy consumption (G = −0.186, *p* < 0.005). Continuous investigation of environmental factors affecting children eating behaviors may help to bring benefits in increasing the effectiveness of health promotion and educational programs.

## 1. Introduction

In Poland, a few decades ago, due to of poor availability of food in the earlier periods of country history, “a plump” child was considered healthy, and the concept of “bigger is better” was widely accepted [1]. Fortunately, this perception is evolving due to existing evidence that overweight and obesity in childhood is associated with a wide range of serious health complications and an increased risk of mortality and morbidity of major chronic diseases including cardiovascular disease, type 2 diabetes, and cancer [2]. At the same time, underweight children tend to become more frequently and severely ill than children with adequate body mass [3]. Underweight is a marker of under-nutrition [4] associated with infections, iron-deficiency anemia, and delay in growth and development among children [3]. According to a study conducted over the years 2007–2009 on a representative sample of Polish pupils (*N* = 17,573, ages 7–18 years, 52.2% girls), the overall incidence of overweight and obesity was 16.4% (18.7% and 14.3% in boys and girls, respectively) and of underweight was 12.0% (10.0% and 13.7% in boys and girls, respectively) [5]. 

Proper nutrition is a key element of maintaining normal body weight in children and adolescents and one of the most important factors determining the optimum development, growth, cognitive functions and overall well-being [6]. It should be accompanied by daily physical activity, which provides fundamental health benefits for children and youth. Higher levels of physical activity are found to be associated with increased physical fitness, reduced body fat, enhanced bone health, and reduced symptoms of depression. It is also related to psychological benefits, better social development, heightened possibility of adoption of other healthy behaviors, and higher academic performance at school [7,8].

In 2017, the Polish National Food and Nutrition Institute published the new food guidelines for children and adolescents. According to this outlines daily diet of children and adolescents should contain three servings of vegetables and two servings of fruit—together at least 400 g. Authors also encourage adults to offer children and adolescents whole grain products and 3–4 glasses of milk every day. Milk can be replaced with natural yoghurt, kefir, and partially by yellow cheese. The guidelines also emphasize the necessity to increase fish consumption, mainly that of marine fish. It should be a part of meals at least twice a week. These recommendations also contain information regarding physical activity, as children should spend at least one hour daily performing moderate to vigorous intensive physical activity [6], in line with the World Health Organization’s (WHO) guidelines [7].

Nationwide research conducted for the Ministry of Sport and Tourism by the Institute of Mother and Child found that in Poland only about 25% of children fulfil this WHO recommendation [9] Typical and officially communicated irregularities existing in the diet of Polish school-age children and adolescents are reported, with too high consumption of sugar, meat, sweetened carbonated beverages, fats, fast food and, at the same time, too low consumption of fruit, vegetables, wholegrain products, dairy, and fish [10]. The prospective cohort research published by the Institute of Mother and Child (*N* = 605; age 13) reported that only 11.4% of participants consumed vegetables more than once a day, while fruit was consumed by 15.5%. According to authors, whole grain bread was consumed daily only by 15.9%, while dairy was consumed by 57.7%. Fish was eaten more than once a week very rarely, that is, by 13.3% of participants [11]. Over last decades a decreased tendency of milk intake among children and adolescents [12] and decreased fish consumption in Poland per capita has been observed [13]. Insufficient consumption of fruit, vegetables, whole grain products, dairy, and fish by Polish children was reported also in other local studies, as cited in discussion section of this article [12,14,15,16,17,18,19,20,21,22,23,24,25,26].

Described consumption behaviors negatively affect children’s health. Sedentary lifestyle is one of the risk factors of chronic diseases because as a result of decreased physical fitness, increased body fat mass and unfavorable cardiovascular and metabolic profiles occur [7]. At the same time, low consumption of fruit and vegetables leads to insufficient supply of some vitamins, minerals, fiber and, in consequence, higher probability of obesity, cardiovascular diseases (CVDs) and stroke [27,28,29]. According to Academy of Nutrition and Dietetics limited consumption of fiber (found also in whole grain cereal products) can lead to an increased risk of developing several chronic diseases, including CVDs, type 2 diabetes and some cancers, and has been associated with excessive body weight [30,31]. Studies also confirmed the nutritional importance of milk products in the human diet. Its adequate consumption may have a role in preventing several chronic conditions such as CVDs, some types of cancers, obesity, and diabetes [32]. Moreover, fish and seafood consumption, thanks to the content of n-3-polyunsaturated fatty acids such as eicosapentaenoic acid and docosahexsaenoic acid, plays a fundamental role in the proper functioning of the brain and the prevention of CVDs [33].

As in the case of adults, the diet of children and adolescents may be influenced by environmental factors such as: the place of residence, dietary trends, wrong perception of their own body, insufficient level of nutrition knowledge of parents, family habits, susceptibility to advertising, stress, and low socio-economic status [34]. Children’s habits are shaped especially by the family environment, therefore parents have an important role in developing healthy dietary patterns of their children, in addition to other elements of healthy lifestyle, that certainly will bring considerable benefits at a later stages of life, giving their offspring the chance at higher quality of life. Lack of nutritional knowledge transfer and poor access to wholesome food products both contribute to perpetuating and strengthening undesirable eating habits among children. In addition, young age is conducive to shaping health behaviors, the positive or negative effects of which can be felt throughout life [35].

The aim of this study was to identify environmental factors which affected children’s health behaviors contributing to the occurrence of diet-related diseases in central Poland. We assessed the influence of place of residence, income, and the level of mothers’ education on children’s behaviors. These socio-economic indicators were chosen since their impact identification may help to better indicate crucial target groups of prevention programs.

## 2. Materials and Methods

A survey was carried out in Poland between September 2016 and March 2017 among mothers whose children were born between 2003 and 2010 and attended classes 1–6 of primary schools. This age group was selected because the beginning of school generally means deterioration of children’s dietary habits, which results in partial independence in the field of food choices [36]. Respondents lived in the city of over 1000 inhabitants and villages. All chosen villages had less than 2000 residents and were located more than 50 km from the closest city with more than 1000 residents. The central region of Poland was selected for the study because it belongs to the province with the highest fertility rate and the highest birth rate [37]. Moreover, it is characterized by Gross Domestic Product (GDP) above the national average [38] and higher level of differentiation of remuneration than the average for total Poland [39].

Data was collected using the semi-structured questionnaire filled in anonymously by mothers during the parents’ meetings organized in schools their children attend. The survey consisted of questions concerning lifestyle and diet-related habits inspired by the Dietary Habits and Nutrition Beliefs Questionnaire published by the Committee of Human Nutrition of the Polish Academy of Sciences Participants [40]. In a question concerning physical activity, mothers were asked whether their child spent time in an active way besides physical education classes at school, for example by engaging in activities such as walking, playing football, and cycling. In case an affirmative answer was given, the mothers were asked to declare the average time the child spent active during the day and what kind of activity was chosen the most often. As further questions concerned whole grain products consumption, examples have been listed for easier identification. Apart from declarations on the frequency of consumption of fruit, dairy products, and fish, mothers were asked to indicate children’s favorite products from these categories. In the case of fish, they were also asked about its cooking method.

To evaluate the impact of environmental factors, the mothers were asked about the place of residence, level of income, education level, number of children and their own and children’s body weight and height. We used the body mass index (BMI) to define anthropometric height/weight characteristics and categorized mothers and children into groups. Based on the declared data, the BMI for mothers was calculated. The adult cut-off points used were 25 kg/m^2^ for overweight and 30 kg/m^2^ for obesity. The BMI for children after calculation was assessed using BMI percentile charts which were constructed using data from recent, large, population representative sample of Polish school-aged children and adolescents (study grant number: PL0080; *N* = 17,573). We used cut-off points recommended by authors: for overweight it was close to the 85th percentile in the case of boys and the 90th percentile in case of girls, while for obesity it was close to the 97th percentile and the 99th percentile, respectively. Using cut-off points recommended by World Health Organization (WHO) according to authors could lead to overestimation of the overweight and obesity rate among Polish girls [41].

For statistical analyses, PAWS Statistics 18 software (SPSS Inc., Hong Kong, China) was used. The chi-squared test (χ^2^) was performed to report on the significance of deviation from expected values for a certain number of observations. Goodman and Kruskal’s gamma (G) was run to determine the association between the analyzed parameters. Statistical significance of differences was estimated at *p* = 0.05.

The study protocol was approved by the Ethics Committee of the Faculty of Human Nutrition and Consumer Sciences of the Warsaw University of Life Sciences (SGGW-WULS) in Warsaw, Poland (No. 11/2017).

## 3. Results

### 3.1. Characteristics of Examined Mothers and Their Children

The questionnaire (response rate 84%) was filled by 892 mothers with a majority being city residents. Most often they had secondary education level, normal body mass (18.5–24.9 kg/m^2^, according to the calculated BMI value) and monthly income per family in the range of 2000–4000 net Polish złoty (PLN) (Table 1).

Mothers living in the city often had a higher education level (49.5% vs. 25.3%) and belonged to the highest net income per family group (37.2% vs. 19.0%). Goodman and Kruskal’s gamma was run to determine the association between the place of residence and the level of mother’s education and net income per family. There was a strong correlation between living in the city and higher education level (G = 0.424, *p* < 0.0005) and between living in the city and higher net income per family (G = 0.386, *p* < 0.0005).

**Scheme 1:**
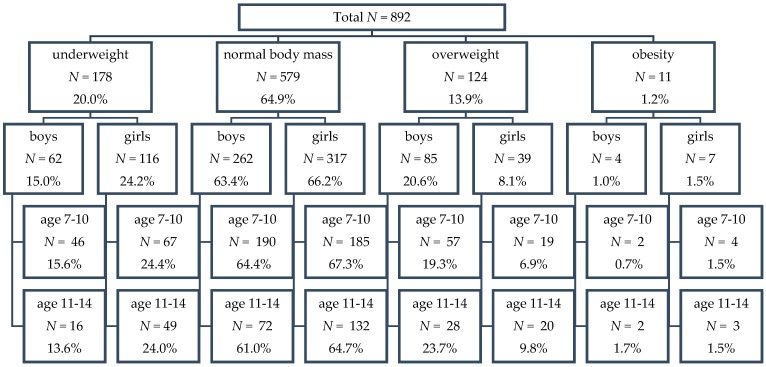
Body mass in population of children (*N* = 892). Percentages may not sum up to 100% due to rounding.

Among group of 892 mothers, we registered 479 mothers of girls and 413 of boys, including 275 girls and 235 boys aged 7–10 years and 204 and 178 aged 11–14 years, respectively. In the children’s group the age median was equal to 10. In an examined group 35.1% of mothers had a child with inadequate body weight, including underweight. A statistically significant association was observed between gender and body mass (χ^2^ = 34.8, *p* < 0.001). Girls were more often underweight than overweight, while boys were the opposite. Extensive body mass was more frequent among elder group for both sexes, while prevalence of underweight was higher amongst younger ones. However, the difference among groups was not statistically significant (Scheme 1).

### 3.2. Physical Activity among Children

**Figure 1:**
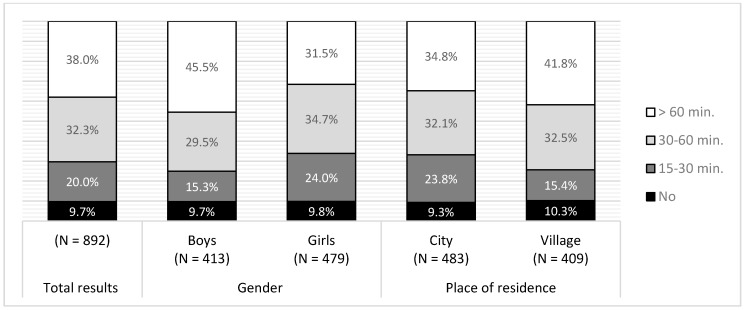
Physical activity in the total surveyed population and sub-groups according to gender and place of residence.

The study assessed the time child spent actively during the day—besides the physical education classes at school. In the surveyed group, most mothers declared that children devoted to this purpose over an hour during the day. Boys spent much more time than girls engaged in physical activity (G = 0.205, *p* < 0.0005). There was also a negative correlation between living in the city and the level of physical activity (G = −0.121, *p* < 0.05) (Figure 1). The most popular declared forms of physical activity were as follows: cycling (64%), football (47%), and swimming (23%).

### 3.3. Vegetable and Fruit Consumption

**Figure 2:**
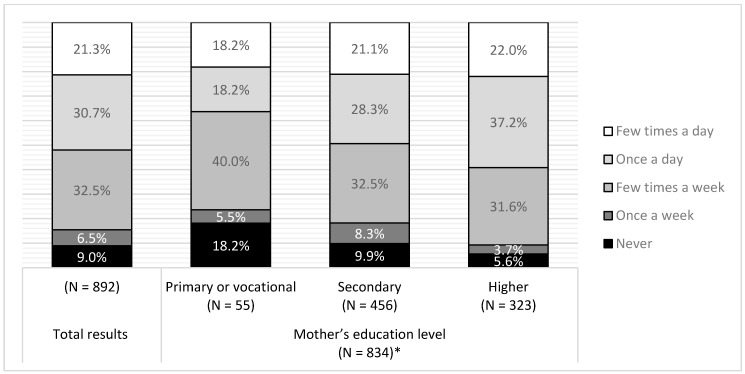
Frequency of vegetable consumption in the total surveyed population and sub-groups according to mother’s education level. * 58 mothers did not declare education level.

**Figure 3:**
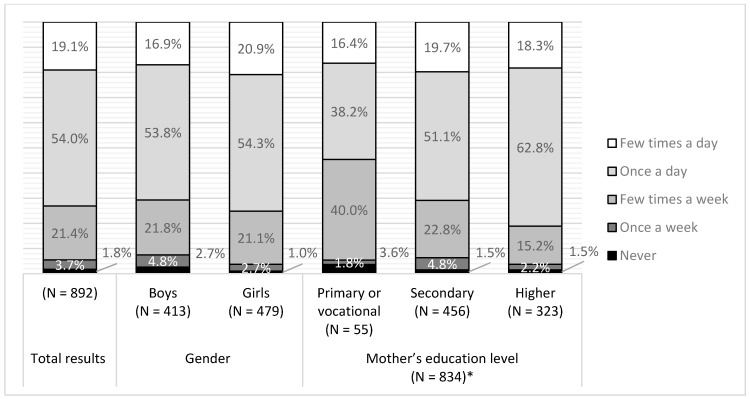
Frequency of fruit consumption in the total surveyed population and sub-groups according to gender and mother’s education level. * 58 mothers did not declare education level.

Based on declarations of mothers, the highest percentage of children ate vegetables a few times a week. There was a positive correlation between vegetable consumption and mothers’ education level (G = 0.167, *p* < 0.0005). Children of mothers with primary or vocational education ate vegetables more rarely than children of mothers with secondary and higher education (Figure 2). The frequency of vegetable consumption decreased with increasing child’s BMI (G = −0.110, *p* < 0.05). The frequency of fruit consumption was higher than that of vegetables. Girls significantly more often consumed fruit than boys (G = 0.116, *p* < 0.05). There was also a positive correlation between fruit consumption and mothers’ education levels (G = 0.155, *p* < 0.005). For fruit we also noticed that the frequency of vegetables consumption significantly decreased with increasing child’s BMI (G = −0.114, *p* < 0.05) (Figure 3). The five most-liked fruits were: apples (82%), bananas (64%), strawberries (21%), pears (20%), and mandarins (17%).

### 3.4. Whole Grain Consumption

**Figure 4:**
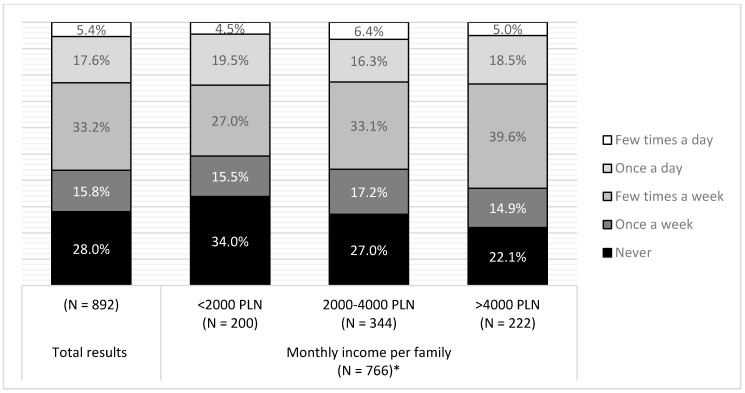
Frequency of whole grain consumption in the total surveyed population and sub-groups according to net monthly income per family. * 126 mothers did not declare monthly income per family. PLN: Polish złoty.

The mothers most often declared that their children consumed whole-grain products less frequently than several times a week, but almost 30% of mothers admitted that their children did not eat them at all. Positive correlation between whole grain consumption and net monthly income level was observed (G = 0.095, *p* < 0.05) (Figure 4).

### 3.5. Dairy and Fish Consumption

**Figure 5:**
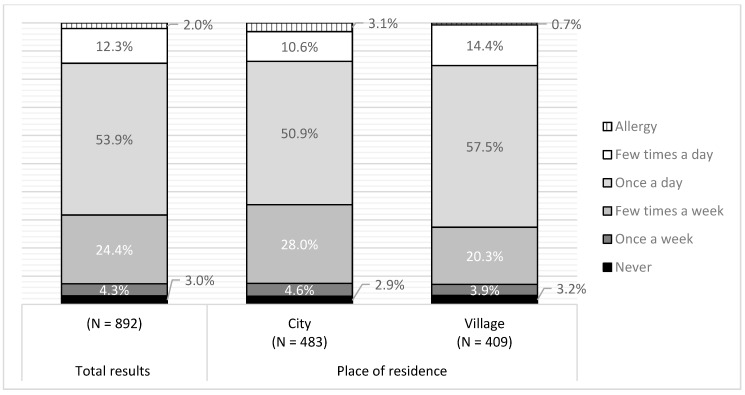
Frequency of dairy consumption in the total surveyed population and sub-groups according to place of residence.

Nearly one-quarter of mothers declared that their children ate dairy products a few times a week, and, more than half, reported that children consumed such products once a day. There was a negative correlation between living in the city and the frequency of dairy consumption (G = −0.186, *p* < 0.005). Mothers in the urban areas more often declared a child’s allergy to cow’s milk protein (Figure 5). The most commonly consumed dairy products indicated by mothers were: cow’s milk (80%), fruit yoghurt (73%), yellow cheese (62%), cocoa (45%), white cheese (34%), and natural yoghurt (27%). In the examined group of children only 10% consumed kefir.

Fish was the one of the food groups that was the most often avoided according to mother’s declarations (21.7%). In the examined group fish was consumed most often only once a week (37.2%), and more frequently than that only by 26.2% (18.3% few times a week and 7.9% once a day). Fifteen percent of examined children ate fish only 1–3 times a month. The fish was served to children mostly in a fried form. Fish based on this cooking method was consumed by 67% of children and the contribution of fish to the diet in this form was the highest in families with the lowest income. Smoked fish (21%), baked fish (13%), and tinned fish meat (12%) were consumed less often. The most commonly consumed fish species were cod (41%), followed by mackerel (25%) and panga (20%). The share of cod and panga varied depending on the level of family income. In the richest families, cod was eaten more frequently (48.2%) and panga less often (13.5%) than in the whole group, while in the poorest families the reverse situation was observed (cod 36%, panga 30.5%).

## 4. Discussion

The Health Behavior in School-aged Children (HBSC) report ranked Poland 10th among all 44 countries surveyed and reported a prevalence of excessive body mass in 11-year-old children equal to 19% [42] Occurrence of overweight and obesity among school children in our research was on a similar level. Our findings are also in line with data reported by other authors for different regions of Poland. In the examined group of primary pupils (*N* = 486, ages 7–10) from south Poland the prevalence of overweight was 14.8%, while obesity prevailed in 1.0% of the cases. [43] In the study group of 1829 children from east Poland excessive body mass concerned 23% children aged 10–12 [21]. In a randomly selected group of 2916 children from different parts of country the results were 15.4% and 3.6% for prevalence of obesity and overweight, respectively [44]. All cited studies, alike our study, reported higher percentage of boys with excessive body mass [5,21,42,43,44].

In the examined population, we noticed surprisingly high percentage of underweight children, especially among girls. Gurzkowska et al., in a large population of 17,427 subjects aged 7–18, received much lower result (11.7%) [45]. Furthermore in smaller research studies, underweight children were not as common as among our sample and ranged from 3% to 15% [5,14,21,43,46].

The Healthy Kids Global Alliance gave Poland the rank D, which means that not more than 40% of children had adequate level of physical activity. Among 37 countries assessed, 10 countries received a better rating than Poland, 10 an equal status, and 16 a poorer status. The total average rank for all countries was equal to D [47]. Our work confirmed this and is in line with other national studies. Analysis published by the National Health Program showed that only 30% of children and adolescents undertook any form of physical activity of the type, frequency, and intensity necessary to meet the physiological needs of the body [48]. This assumption is also supported by results of the HBSC report—27.6% of pupils aged 11–12 years were engaged in physical activity for an hour a day and in the age category 13–14 years the value was −23.4% [49].

In our study boys spent significantly more time in an active way than girls. Other authors also confirmed significant differences based on gender [9,50]. Despite the fact that boys in our examined group spent more time in an active way, they also more often had an excessive body mass. It could be a result of an unbalanced diet, i.e., higher in comparison to girls’ consumption of sweet beverages [49,51,52,53]. In our study, children from villages were more active than those living in the city, which was in line with results obtained of surveys by children from South Poland [54], East Poland [55] and Ukrainian students [56]. During literature review, we also found an opposite result reported for Polish children, however, in this work the author compared villages to small towns [57]. The results of a 12-country study showed that children from Colombia, Kenya, and Portugal had a lower probability of meeting WHO activity guidelines if their mothers had attained higher education level. This research and other authors concluded that there is a negative association between parental education level and child’s physical activity in lower economic status countries, while there is a positive relation between these factors in higher economic status countries [58,59,60,61]. In the work concerning Polish pupils aged 13–15 (*N* = 1706), the level of activity was significantly higher among children of mothers with higher education [62]. Our study and results from East Poland [63] did not report such an influence.

A cross-sectional survey, which described fruit and vegetable intake of 11-year-old children, showed that this consumption was below recommended levels (<400 g per day) among the school children in all 10 examined countries (Bulgaria, Finland, Germany, Greece, Iceland, Norway, Portugal, Slovenia, Sweden, and the Netherlands). The research also showed that vegetable intake was lower than fruit intake and that there is a need for promotional activities to improve fruit and vegetable consumption in this age group [64]. A rarer than recommended frequency of these products’ consumption was also observed in our work and by other authors. Wolnicka’s and Jaczewska-Schuetz’s (*N* = 380; ages 11–13) claimed that fruit and vegetables are not eaten daily by 35.8% of pupils [14]. Stefańska et al. noted that the recommended frequency of consumption (more than once a day) of vegetables was declared by only 15% of girls and 12% boys aged 10–12 years [21]. Cieślik et al. observed that nearly 20% of the children did not eat vegetables in general [24].

The influence of gender on the amount of fruit and vegetable consumption by children was examined in our study. A review of Rasmussen et al. reported that 22 out of 49 studies documented that girls ate fruit and vegetables more frequently than boys. In 18 studies no differences were observed in fruit and vegetables consumption between the genders, while in four studies a higher consumption amongst boys was observed [65]. In our work, girls ate fruit significantly more frequently than boys. The consumption of vegetables was at the same level in boys and girls. The only environmental factor that was associated with more frequent consumption of both fruit and vegetables was mothers’ education levels. Positive influence of higher maternal education level on children’s fruit and vegetable consumption was reported previously by other authors [66,67,68,69,70,71].

Similar to our results, those for whole grain product consumption were reported in other works in Poland. In the examined population, among teenagers aged 14–16 (*N* = 400) only 25% of subjects ate wholegrain cereal products daily [15]. In other research involving pupils aged 11–13 (*N* = 1100) whole grain bread was eaten reluctantly and 31% of respondents declared its consumption less often than once a week. At the same time, dark bread was eaten daily only by 11% respondents [20]. Further, in the population of children aged 10–15 (*N* = 1829) brown bread was eaten a few times a week by about 40% of pupils [21]. Very low consumption of whole grain cereals and bread was also reported among pupils aged 13–16 (*N* = 202)—only 6.2% of them consumed whole-grain cereal, bread, and brown bread [22]. Similar results were reported by other authors [23,24,72]. Low consumption of whole grains was documented also for French children (6–8 g per day) [73], while moderate consumption was reported for North American pupils (9.4–14.4 g per day) [74,75] and higher consumption for U.K., Irish, and German pupils (13.0 g, 18.60 g, and 24.3 g, respectively) [76,77,78]. During the literature review, we found other papers that also documented a correlation between higher income level and frequency of whole grain consumption in Poland [79].

The results of our research showed that more than half of the examined group consumed milk products daily, but only every ninth child consumed dairy products several times a day. This allows us to suspect that the examined children did not consume the 3–4 portions of dairy products recommended by the Polish National Food and Nutrition Institute. As in our work, research among urban pupils aged 9–13 (*N* = 100) from North Poland demonstrated that children most often consumed dairy products once a day (65%) [25]. Other data on the frequency regarding the consumption of dairy products among 50 pupils aged 11–14 from East Poland showed similar results—milk and milk products were consumed daily by 64% [26]. Like in our study, the most popular products in children’s diet in both cited works were milk, fruit yoghurt and yellow cheese [25,26]. Lower than recommended consumption of dairy products (Polish National Food and Nutrition Institute) was also demonstrated in the works of other researchers [12,16,17]. Dairy consumption seems to be insufficient not only in Poland. An international report showed that daily average intake of these products was equal to about one portion a day for children residing in France, Ireland, Germany, and the United Kingdom. Higher dairy consumption was reported only in Spain, Singapore, and Australia [80].

In our presented study, we found significant influence of place of residence on dairy product intake. More frequent consumption was reported among rural children. Opposite result to ours was obtained for respondents aged 13 to 75 (*N* = 9339) from six macro-regions of Poland, as it was shown that rural residents ate milk and milk products less frequently [81].

Fish consumption in Poland is generally low in all age groups. According to research which examined fish consumption patterns in children and their mothers in 17 EU countries, Polish families had rather low consumption of fish, at similar levels to those of Switzerland, the Czech Republic, Hungary, Romania, Slovenia, and Slovakia. The opposite situation was reported for Belgium, Denmark, Spain, Portugal, and Sweden [82]. The situation in Poland has not improved for years as our results are in line with the studies conducted about 10 years ago. A study among pupils aged 11–19 (*N* = 2200) showed that only 35% of school children ate fish once a week and 11% ate fish more often [18], while in a study concerning pupils aged 7–19 (*N* = 899) the results were 40% and 18%, respectively [19]. As in our research, school youth according to other studies consumed mainly breaded fried fish and partly or highly manufactured products [83,84].

## 5. Conclusions

The majority of examined children consumed vegetables, fruit, whole grain products, dairy products, and fish at a lower frequency than that recommended by the Polish National Food and Nutrition Institute. Thus, it is necessary to continue and strengthen nutritional education in primary schools as such diet patterns, if coexisting with low physical activity, may increase the risk of diet-related diseases. Educational programs targeted at schools should also focus on parents. A greater availability of products consumed in insufficient quantities, particularly whole grains and fish, should be ensured in school canteens, and could provide a chance for their consumption for children from poorer families. We believe that further investigation of environmental factors affecting children eating behaviors may help to bring benefits in increasing the effectiveness of health promotion and educational programs.

## Figures and Tables

**Table 1:** Characteristics of the respondent sample (*N* = 892).

Environmental Factors		*N*	%
(1). Place of residence	City	483	54.1
	Village	409	45.9
(2). Mother’s education level	Basic and vocational education	55	6.2
	Secondary education	456	51.1
	Higher education	323	36.2
	Not declared	58	6.5
(3). Mother’s body mass (according to BMI)	Underweight	34	3.8
	Normal body mass	542	60.8
	Overweight	183	20.5
	Obese	45	5.0
	Not declared	88	9.9
(4). Net income per family *	<2000 PLN	200	22.4
	2000–4000 PLN	344	38.6
	>4000 PLN	222	24.9
	not declared	126	14.1
(5). Number of children in the family	1	196	22.0
	2	471	52.8
	3	156	17.5
	4+	69	7.7

* Not all mothers declared income, body mass and education level. PLN: Polish złoty; BMI: body mass index.
